# Afar triple junction triggered by plume-assisted bi-directional continental break-up

**DOI:** 10.1038/s41598-018-33117-3

**Published:** 2018-10-03

**Authors:** Alexander Koptev, Taras Gerya, Eric Calais, Sylvie Leroy, Evgueni Burov

**Affiliations:** 1 Sorbonne Université, CNRS, Institut des Sciences de la Terre de Paris (ISTeP), Paris, France; 2 Department of Geosciences, University of Tübingen, Tübingen, Germany; 3 ETH-Zurich, Institute of Geophysics, Sonnegstrasse 5, 8092 Zurich, Switzerland; 4 Ecole Normale Supérieure, Department of Geosciences, PSL Research University, CNRS UMR 8538 Paris, France

## Abstract

Divergent ridge-ridge-ridge (R-R-R) triple junctions are one of the most remarkable, yet largely enigmatic, features of plate tectonics. The juncture of the Arabian, Nubian, and Somalian plates is a type-example of the early development stage of a triple junction where three active rifts meet at a ‘triple point’ in Central Afar. This structure may result from the impingement of the Afar plume into a non-uniformly stressed continental lithosphere, but this process has never been reproduced by self-consistent plume-lithosphere interaction experiments. Here we use 3D thermo-mechanical numerical models to examine the initiation of plume-induced rift systems under variable far-field stress conditions. Whereas simple linear rift structures are preferred under uni-directional extension, we find that more complex patterns form in response to bi-directional extension, combining one or several R-R-R triple junctions. These triple junctions optimize the geometry of continental break-up by minimizing the amount of dissipative mechanical work required to accommodate multi-directional extension. Our models suggest that Afar-like triple junctions are an end-member mode of plume-induced bi-directional rifting that combines asymmetrical northward pull and symmetrical EW extension at similar rates.

## Introduction

Triple junctions, places where the boundaries of three tectonic plates come together^1^, are a peculiar geometry that has been of research interest since the birth of plate tectonics. Under the approximation of rigid plates, the stability of triple junctions is defined by the relative plate velocity vectors^1,2^. Ridge-ridge-ridge (R-R-R) triple junctions, in particular, are stable for all extensional rates and ridge configurations^1^. Beyond this kinematic description, the physical mechanism that controls the initiation and evolution of triple junctions is still not well understood^3–5^. It has been proposed that they form as a result of axisymmetric dome uplifts expected to form above mantle upwellings, which cause lithospheric breakup along three rifts striking 120° to each other, as required to minimize mechanical work^3^. Although old physical experiments showed the development of linear graben structures caused by diapiric uprise of viscous material with no external lateral extension^6^, recent numerical experiments reveal that mantle plume impingement on a rheologically realistic lithosphere not subjected to far-field stresses may only result in axisymmetric domal-shaped features with multiple radiating rifts^7^. In contrast, under uni-directional extension, numerical models usually evolve into linear rift-like structures and do not show multi-branch junctions^7–9^. Therefore, the initiation and evolution of R-R-R triple junctions likely involves more complex boundary conditions, possibly bi-directional^5^. R-R-R triple junctions have been modelled for relatively simple conditions of oceanic plate break-up^4,5^, but their initiation in continental lithosphere under realistic multi-directional boundary conditions remains unaddressed.

## A Type-Locale for Triple Junctions: The Afar Region

**Figure 1:**
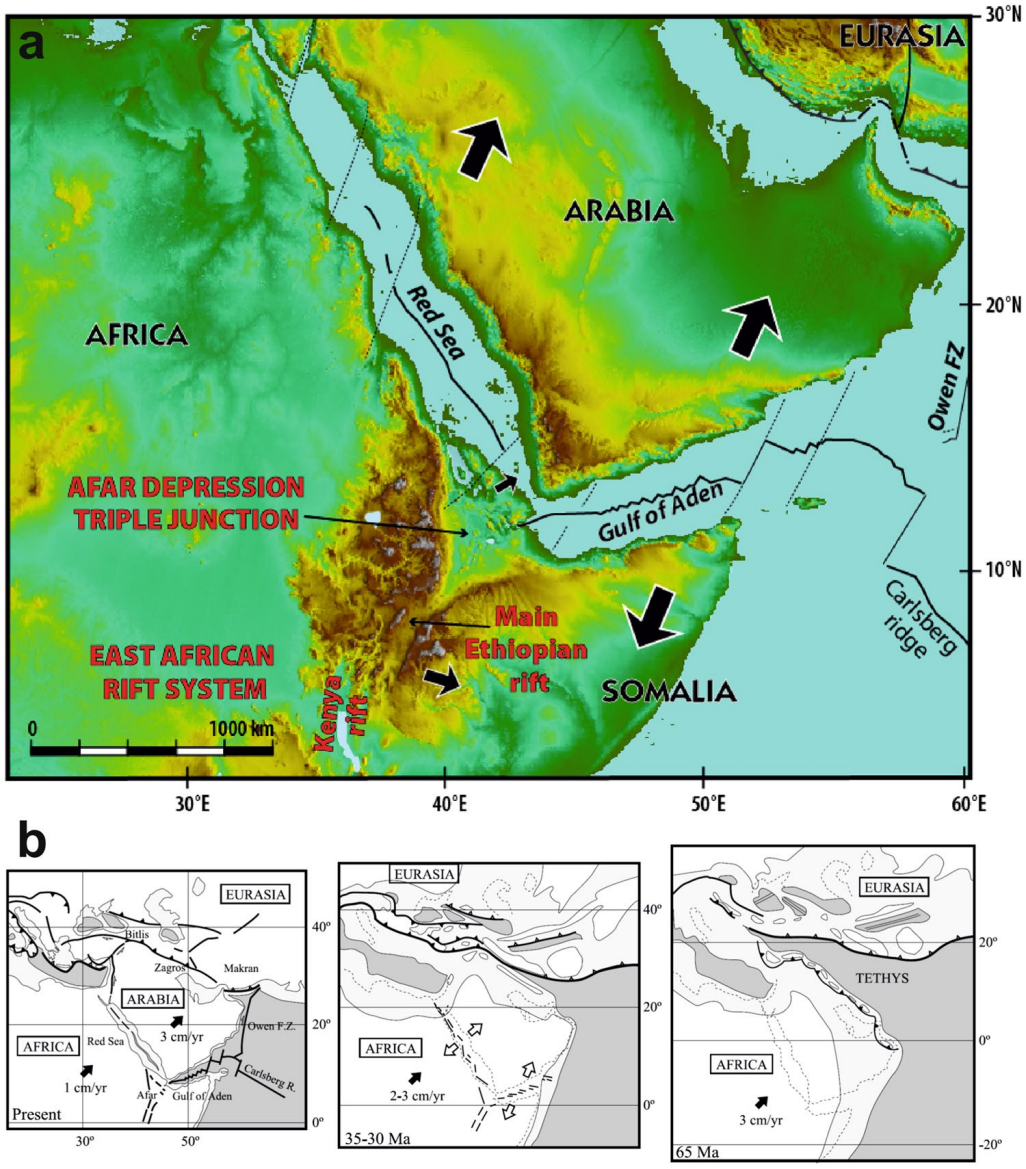
Tectonic framework and evolution of the Arabian, Nubian, and Somalian plates. (**a**) Present-day tectonic setting of eastern Africa and Arabia. (**b**) Tertiary tectonic evolution of the Africa-Arabia-Eurasia system^18^.

The Afar depression, the only emerged R-R-R triple junction on Earth, is a type-locale for such structures (Fig. 1). It marks the juncture between the Red Sea, Gulf of Aden, and Main Ethiopian rifts, which accommodate the relative motion between the Arabian, Nubian, and Somalian plates^10,11^. Rifting in the Red Sea and Gulf of Aden started in the Late Eocene - Early Oligocene^12–15^, ultimately resulting in Africa-Arabia break-up in the Early Miocene^16,17^. Divergence between Arabia and Africa has been attributed to extensional stresses imparted by the slab-pull force that developed along the subduction boundary between the Eurasian and Afro-Arabian plates^18^. The Mediterranean and Biltis segments of that plate boundary progressively transitioned to continental collision between 30 and 20 Ma, with a slow-down of the Africa-Eurasia convergence^19^ whereas active subduction continued along the remaining eastern segment of the Neo-Tethys^20^. The eastward closing of that ocean^21^ led to a gradual westward decrease of slab pull forces from the Makran subduction to the collisional segment (Fig. 1b). The early collision stage coincides with the eruption of voluminous flood basalts linked to the emplacement of the Afar plume at ~30 Ma^22^. Thus, the Africa-Arabia separation was likely favoured not only by appropriate intraplate stress generated by lateral variation of slab-pull force along the Neo-Tethys subduction, but also by the thermal weakening effect of the plume impingement that facilitated strain localization inside the Afro-Arabian plate^18^.

At the same time, anomalous positive topography started to form in East Africa^23^, with the first evidence of doming in Ethiopia at ~30–40 Ma likely related to the Afar plume emplacement^22^. The current long-wavelength elevation of eastern and southern Africa, dynamically supported by the African Superplume, a broad low-velocity seismic anomaly imaged in the lower mantle^24,25^, is acquired at ~10 Ma^23^ although the exact timing and dynamics of uplift in the Horn of Africa are still controversial^26–28^. The resulting lateral gradients of gravitational potential energy generate EW-directed extensional deviatoric stresses corresponding to forces on the same order as slab pull forces when integrated over the thickness of the lithosphere^29^. Given that the African continent is surrounded mostly by oceanic ridges, far-field extension can only be driven by a combination of lateral gradients of gravitational potential energy and viscous coupling with horizontal mantle flow at the base of the lithosphere^29–31^. Recent results of spherical shell finite element modelling of regional stress and strain field in Africa has shown that compressional stresses applied at the mid-ocean ridges surrounding Africa result in rift-perpendicular deviatoric extension in the East African rift valleys that, being combined with upwelling mantle plume(s), leads to localization of extensional deformation and explains the initial lithosphere break-up in the East Africa and the Afar^32^. Likewise, recent 3D thermo-mechanical deformation models show that pre-stressed lithosphere subjected to EW extension is a necessary initial condition for plume-induced localized rifts to develop in the central part of the East African Rift^8,9,33^.

We note that the detailed reproduction of the the timing of triple junction development is beyond the scope of this paper. We do not target to include such aspects as a delayed rifting in Northern Ethiopia with respect to that in the Red Sea and Gulf of Aden^34–38^ or a westward propagation of the Gulf of Aden opening in the Miocene due to lateral propagation of the Sheba Ridge from the Indian Ocean into the African continent^15,39,40^. In contrast, our main objective is to quantify the general consequences of thermal and buoyancy-driven mechanical effects of the plume head in the context of laterally homogenous lithosphere subjected to bi-directional, asymmetric far-field stresses. To this purpose we first present the generic features of the experiments, followed by a comparison of our model inferences with observed present-day configuration of break-up zones in the Afar triple junction.

## Modelling Approach and Results

The Afar triple junction forms in a context where a plume interacts with a continental lithosphere subjected to bi-directional far-field forcing that combines northward pull from the Neo-Tethys slab subduction and EW extension. We use this configuration to design a model geometry that we implement into high-resolution 3D thermo-mechanical numerical calculations using the viscous-plastic I3ELVIS code^41^, which is based on a combination of a finite difference method with a marker-in-cell technique (see Methods). We use a 1500 × 1500 × 635 km rectangular model domain with 297 × 297 × 133 nodes that offers a spatial resolution of ca. 5 × 5 × 5 km per grid cell. The laterally homogenous lithosphere consists of a bi-layer, 36-km-thick crust and 114-km-thick lithospheric mantle. We initiate a mantle plume by seeding a 200 km-radius hemispheric temperature anomaly at the base of the upper mantle, 300 K warmer than the surroundings (Supplementary Fig. 1). In contrast to previous studies that use an arbitrary pre-defined triple junction geometry^4,42^, we impose no pre-existing structuration within the crust or lithospheric mantle in order to investigate the spontaneous initiation of a divergent triple junction in response to the simultaneous action of the mantle plume and far-field extensional stresses. We simulate tectonic forcing by applying a constant divergent velocity normal to the “eastern” and “western” model boundaries combined with laterally varying pull along the northern side of the model (Supplementary Fig. 1). We perform three groups of models with EW extension half-rates of 0, 3, and 6 mm/yr (Supplementary Table 1). These boundary velocities are derived from the Neogene kinematics of the Nubia-Somalia plate system^43^. Each group consists of six experiments that use the following kinematic boundary conditions along northern side of model domain: free-slip (i.e. absence of northward pull), constant northward pull of 4 mm/yr, and northward pull linearly increased from west to east from 0–6 mm/yr to 3–6 mm/yr, 6–12 mm/yr, and 12–18 mm/yr (Supplementary Table 1). The laterally-varying northward pull is meant to mimic the geodynamics of the northern convergent margin of the Afro-Arabian plate at the time of the Afar plume impingment^18^ (Fig. 1b).

**Figure 2:**
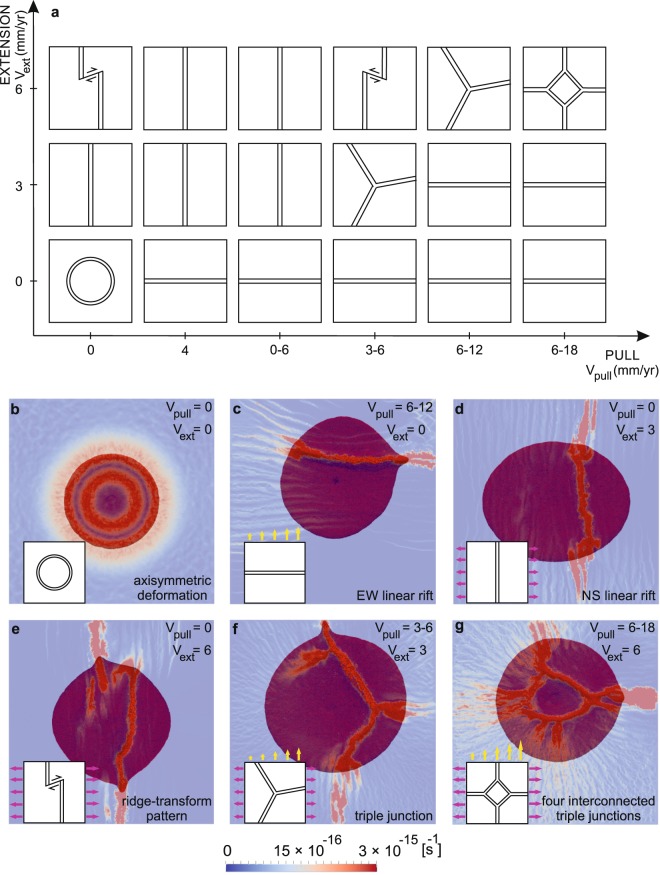
Deformation modes resulting from the 3D experiments. (**a**) Schematic representation of deformation modes as a function of the applied velocity boundary conditions (see also Supplementary Fig. 2). Note the NS trend of localized extensional structures in cases where EW extension dominates and ~EW rift orientation for experiments where northward pull dominates. Close values of extension and pull favour the development of R-R-R triple junctions. (**b**–**g**) Top view of the most representative experiments for (**b**) axisymmetric mode of deformation, (**c**) EW and (**d**) NS linear rift, (**e**) ridge-transform pattern, (**f**) triple junction, and (**g**) four interconnected triple junctions. Blue to red colours indicate crustal strain rate distribution at the moment of break-up. The plume material is shown in dark red.

The results summarized in Fig. 2 show that far-field extensional stresses play a key role in the style of system development. A non-pre-stressed lithosphere expectedly results in axisymmetric surface deformation in response to radial lateral spreading of the plume head while radial crustal cracks are not distinguishable because of the limited resolution of the model (Fig. 2b). In contrast, the presence of any far-field stress triggers the development of non-axisymmetric features that are governed by the value and orientation of the boundary velocities applied. EW linear rifts (Fig. 2c) occur in experiments where northward pull dominates EW extension. On the contrary, faster EW extension leads to continental break-up localized along extension-perpendicular (i.e. NS oriented) structures (Fig. 2d). In certain cases, two en échelon overlapping spreading zones trending in a NS direction are connected by a >200 long orthogonal transform zone, forming a complex transform-ridge pattern (Fig. 2e). An Afar-like triple junction end-member (Fig. 2f) develops when EW extension (3 mm/yr) and northward pull (3–6 mm/yr) are close. Note that full EW extension of 6 mm/yr in this model is consistent with present-day geodetic estimates for the northernmost segment of the Main Ethiopian rift^44^. We observe that the synchronous increase in both EW extension (up to 6 mm/yr) and northward pull (up to 6–12 mm/yr) does not change significantly this pattern (Supplementary Fig. 2e). A further augmentation of the northward pull (up to 6–18 mm/yr) results in a ring-like structure with four radial axes, which are combined in a series of four interconnected triple junctions (Fig. 2g).

Four main rifting modes can thus be distinguished: (i) axisymmetric deformation (Fig. 2b), (ii) linear rift(s) stretched in EW to WNW-ESE (Fig. 2c) or NS to NW-SE (Fig. 2d) directions, (iii) ridge-transform pattern (Fig. 2e) and (iv) single triple junction (Fig. 2f) or four interconnected triple junctions resulting in a ring-like structure (Fig. 2g). Despite the theoretical stability of R-R-R triple junctions^1^, their spontaneous initiation in numerical experiments appears to be controlled by far-field tectonic forcing. In most our experiments, extensional deformation is localized along one or two linear structures oriented perpendicularly to the dominant forces, whereas more complex patterns such as divergent triple junctions occur only within a limited range of extension/pull combinations (Fig. 2a; Supplementary Fig. 2).

We extracted shear heat production (*H*_*s*_) from each successive stage of model 10 (Supplementary Fig. 2). *H*_*s*_ is a measure of the dissipation of mechanical energy during irreversible non-elastic (e.g., viscous) deformation (Supplementary Fig. 3). We observe two episodes of rapid decrease of mechanical work in the system, one at 0–10 Myr when upper crustal deformation dominates the initial stage of the model evolution, the other at 40–55 Myr as the model transitions from single break-up axis to the final triple junction configuration. We interpret this as showing that a triple junction provides the optimal geometrical structure for continental break-up under the conditions of model 10 – that most resembles the Afar triple junction – as it maximizes the rate of decrease of the dissipative mechanical work required for multi-directional extension. The stability of Afar-like triple junctions thus satisfies the principle of minimum mechanical dissipative work^5^.

## Implications for the Afar Triple Junction

**Figure 3:**
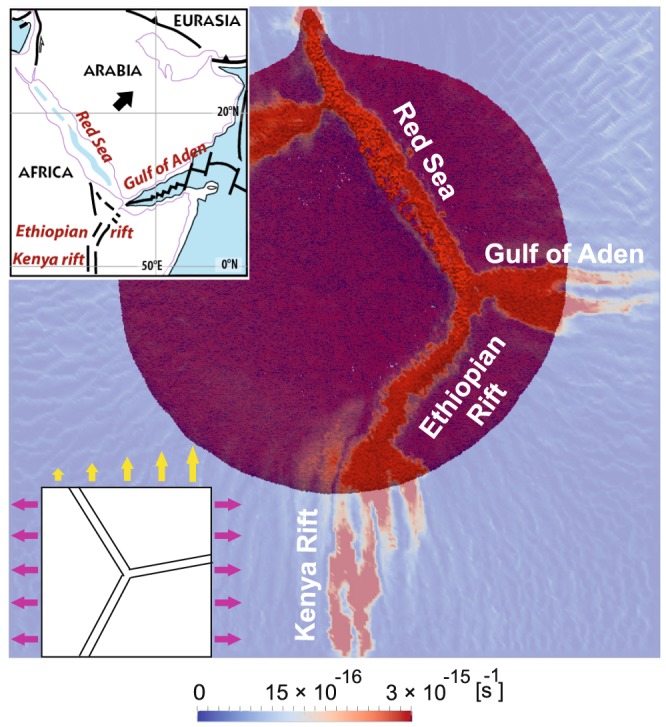
Triple junction model. This experiment (extension half rate of 3 mm/yr combined with irregular northward pull of 3–6 mm/yr; see Fig. 2f) reproduces the first-order structures of Afro-Arabian rift system from the Red Sea to the Kenyan Rift (top left insert).

The configuration of break-up zones in the triple junction experiment shown on Fig. 2f mimics the geometry of the Afar triple junction quite well, as shown on Fig. 3, despite the relative simplicity of the initial model setup: a NW-SE-trending northern rift branch (similar to the Red Sea rift) joins two other rift branches, one trending ENE (Gulf of Aden rift), the other trending NE-SW (Main Ethiopian rift). The latter propagates southward forming an extension-perpendicular, NS-trending rift similar to the eastern branch of the Central East African rift system (i.e., Kenyan rift). However, it is not straightforward to extrapolate the model results to south of the Main Ethiopian rift where the system is complicated by the stress transmission across the strong Tanzanian craton to the western branch^45^ and by the presence of low velocity anomaly in the upper mantle beneath Kenya and northern Tanzania^46–48^ that controls along-axis rift variations in the eastern branch^49^. Also, an additional rift arm west of the Red Sea that trends WSW-ENE does not find its reflection in nature.

Thus, this model reproduces the first-order structures of the Afro-Arabian rift system from the Red Sea to the Kenyan Rift in the simple context of an initially homogenous lithosphere due to the thermal impact of the active mantle plume and its buoyancy-driven flow combined with bi-directional, asymmetric far-field stresses. The presence of pre-existing linear zones of weakness, proposed to explain the formation of the Red Sea^50,51^ and Gulf of Aden^52^, appears not to be mandatory for deformation to localize and, ultimately, lead to the present configuration of the Afar triple junction.

## Methods

We produced the simulations presented in this contribution using numerical code I3ELVIS that is based on a combination of a finite difference method with a marker-in-cell technique^41,53^. This parallel 3D code allows for non-diffusive numerical simulation of multiphase flow in a rectangular fully staggered Eulerian grid^54^. The momentum equations are solved in the form of Stokes flow approximation. The components of the deviatoric stress tensor are calculated using the viscous constitutive relationship between stress and strain rate for a compressible fluid. The mechanical equations are fully thermodynamically coupled with heat conservation equations accounting for mineralogical phase changes, adiabatic, radiogenic and frictional internal heating sources.

Non-newtonian viscous-plastic rheologies are implemented via evaluation of the effective viscosity of the material. The material deforms according to Newtonian diffusion creep and power law dislocation creep. In addition, Peierl’s plasticity applies when stress reaches a specific limit (“Peierl’s stress”) that characterizes transition to plastic failure^55,56^. The contributions from dislocation, diffusion and Peierls creep are taken into account via computation of inverse average viscosity. The ductile rheology is combined with a brittle/plastic rheology to yield an effective visco-plastic rheology with a Drucker-Prager yield criterion. The visco-plastic rheology is assigned to the model by means of a “Christmas tree”-like criterion, where the rheological behaviour depends on the minimum viscosity attained between the ductile and brittle/plastic fields^57–59^.

The free surface topography is reproduced using the ‘sticky air’ technique enhanced by the introduction of a high-density marker distribution in the near-surface zone.

The implicit parallel numerical scheme uses an indirect multigrid solver^53^, that permits to accelerate 3D calculations.

In-depth description of the computer code I3ELVIS is provided in the book by T. Gerya^53^.

## Electronic supplementary material

Supplementary information

## References

[1] McKenzie DP, Morgan W (1969). Evolution of triple junctions. Nature.

[2] Patriat P, Courtillot V (1984). On the stability of triple junctions and its relation to episodicity in spreading. Tectonics.

[3] Burke K, Dewey JF (1973). Plume-generated triple junctions: key indicators in applying plate tectonics to old rocks. The Journal of Geology.

[4] Dordevic M, Georgen J (2016). Dynamics of plume–triple junction interaction: Results from a series of three-dimensional numerical models and implications for the formation of oceanic plateaus. Journal of Geophysical Research: Solid Earth.

[5] Gerya, T. & Burov, E. Nucleation and evolution of ridge-ridge-ridge triple junctions: Thermomechanical model and geometrical theory. *Tectonophysics*, 10.1016/j.tecto.2017.10.020 (2017).

[6] Mulugeta G (1985). Dynamic models of continental rift valley systems. Tectonophysics.

[7] Burov E, Gerya T (2014). Asymmetric three-dimensional topography over mantle plumes. Nature.

[8] Koptev A, Calais E, Burov E, Leroy S, Gerya T (2015). Dual continental rift systems generated by plume-lithosphere interaction. Nature Geoscience.

[9] Koptev A (2016). Contrasted continental rifting via plume-craton interaction: Applications to Central East African Rift. Geoscience Frontiers.

[10] McKenzie DP, Davies D, Molnar P (1970). Plate tectonics of the Red Sea and East Africa. Nature.

[11] McClusky S, Reilinger R, Mahmoud S, Sari DB, Tealeb A (2003). GPS constraints on Africa (Nubia) and Arabia plate motions. Geophysical Journal International.

[12] Roger J, Platel JP, Cavelier C, Bourdillon-de-Grisac C (1989). Données nouvelles sur la stratigraphie et l’histoire géologique du Dhofar (Sultanat d’Oman). Bulletin de la Société géologique de France.

[13] Ghebreab W (1998). Tectonics of the Red Sea region reassessed. Earth-Science Reviews.

[14] Watchorn F., Nichols G. J., Bosence D. W. J. (1998). Rift-related sedimentation and stratigraphy, southern Yemen (Gulf of Aden). Sedimentation and Tectonics in Rift Basins Red Sea:- Gulf of Aden.

[15] Leroy S (2012). From rifting to oceanic spreading in the Gulf of Aden: a synthesis. Arabian Journal of Geosciences.

[16] Joffe S, Garfunkel Z (1987). Plate kinematics of the circum Red Sea - a re-evaluation. Tectonophysics.

[17] Mohriak WU, Leroy S (2013). Architecture of rifted continental margins and break-up evolution: insights from the South Atlantic, North Atlantic and Red Sea–Gulf of Aden conjugate margins. Geological Society, London, Special Publications.

[18] Bellahsen N, Faccenna C, Funiciello F, Daniel JM, Jolivet L (2003). Why did Arabia separate from Africa? Insights from 3-D laboratory experiments. Earth and Planetary Science Letters.

[19] McQuarrie N., Stock J. M., Verdel C., Wernicke B. P. (2003). Cenozoic evolution of Neotethys and implications for the causes of plate motions. Geophysical Research Letters.

[20] Jolivet L, Faccenna C (2000). Mediterranean extension and the Africa-Eurasia collision. Tectonics.

[21] Decourt, J., Ricou, L. E. & Vrielynck, B. Atlas Tethys Palaeoenvironmental Maps, BEICIP-FRANLAB. *Gauthier-Vollars, Paris* **14** (1993).

[22] Hofmann C (1997). Timing of the Ethiopian flood basalt event and implications for plume birth and global change. Nature.

[23] Paul JD, Roberts GG, White N (2014). The African landscape through space and time. Tectonics.

[24] Van der Hilst RD, Widiyantoro S, Engdahl ER (1997). Evidence for deep mantle circulation from global tomography. Nature.

[25] Lithgow-Bertelloni C, Silver PG (1998). Dynamic topography, plate driving forces and the African superswell. Nature.

[26] Pik R, Marty B, Carignan J, Lavé J (2003). Stability of the Upper Nile drainage network (Ethiopia) deduced from (U–Th)/He thermochronometry: implications for uplift and erosion of the Afar plume dome. Earth and Planetary Science Letters.

[27] Gani ND, Gani MR, Salam A (2007). Blue Nile incision on the Ethiopian Plateau: Pulsed plateau growth, Pliocene uplift, and hominin evolution. GSA Today.

[28] Sembroni A, Faccenna C, Becker TW, Molin P, Abebe B (2016). Long-term, deep-mantle support of the Ethiopia-Yemen Plateau. Tectonics.

[29] Stamps DS, Flesch LM, Calais E, Ghosh A (2014). Current kinematics and dynamics of Africa and the East African Rift System. Journal of Geophysical Research: Solid Earth.

[30] Coblentz DD, Sandiford M (1994). Tectonic stresses in the African plate: Constraints on the ambient lithospheric stress state. Geology.

[31] Stamps DS, Iaffaldano G, Calais E (2015). Role of mantle flow in Nubia-Somalia plate divergence. Geophysical Research Letters.

[32] Min G, Hou G (2018). Geodynamics of the East African Rift System ~30 Ma ago: A stress field model. Journal of Geodynamics.

[33] Koptev A, Cloetingh S, Gerya T, Calais E, Leroy S (2018). Non-uniform splitting of a single mantle plume by double cratonic roots: Insight into the origin of the central and southern East African Rift System. Terra Nova.

[34] Wolfenden E, Ebinger C, Yirgu G, Deino A, Ayalew D (2004). Evolution of the northern Main Ethiopian rift: birth of a triple junction. Earth and Planetary Science Letters.

[35] Bonini Marco, Corti Giacomo, Innocenti Fabrizio, Manetti Piero, Mazzarini Francesco, Abebe Tsegaye, Pecskay Zoltan (2005). Evolution of the Main Ethiopian Rift in the frame of Afar and Kenya rifts propagation. Tectonics.

[36] Rooney, T., Furman, T., Bastow, I., Ayalew, D. & Yirgu, G. Lithospheric modification during crustal extension in the Main Ethiopian Rift. *Journal of Geophysical Research: Solid Earth* **112** (2007).

[37] Keranen K, Klemperer SL (2008). Discontinuous and diachronous evolution of the Main Ethiopian Rift: Implications for development of continental rifts. Earth and Planetary Science Letters.

[38] Stab M (2016). Modes of rifting in magma-rich settings: Tectono-magmatic evolution of Central Afar. Tectonics.

[39] Bosworth W, Huchon P, McClay K (2005). The Red Sea and Gulf of Aden basins. Journal of African Earth Sciences.

[40] Fournier, M. et al. Arabia-Somalia plate kinematics, evolution of the Aden-Owen-Carlsberg triple junction, and opening of the Gulf of Aden. *Journal of Geophysical Research: Solid Earth* **115** (2010).

[41] Gerya TV, Yuen DA (2007). Robust characteristics method for modelling multiphase visco-elasto-plastic thermo-mechanical problems. Physics of the Earth and Planetary Interiors.

[42] Georgen JE (2011). Lithospheric control on the spatial pattern of Azores hotspot seafloor anomalies: Constraints from a model of plume-triple junction interaction. Geophysical Research Letters.

[43] DeMets C, Merkouriev S (2016). High-resolution estimates of Nubia–Somalia plate motion since 20 Ma from reconstructions of the Southwest Indian Ridge, Red Sea and Gulf of Aden. Geophysical Journal International.

[44] Saria E, Calais E, Stamps DS, Delvaux D, Hartnady CJH (2014). Present-day kinematics of the East African Rift. Journal of Geophysical Research: Solid Earth.

[45] Nyblade AA, Brazier RA (2002). Precambrian lithospheric controls on the development of the East African rift system. Geology.

[46] Nyblade AA, Owens TJ, Gurrola H, Ritsema J, Langston CA (2000). Seismic evidence for a deep upper mantle thermal anomaly beneath east Africa. Geology.

[47] Chang SJ, Van der Lee S (2011). Mantle plumes and associated flow beneath Arabia and East Africa. Earth and Planetary Science Letters.

[48] O’Donnell JP, Adams A, Nyblade AA, Mulibo GD, Tugume F (2013). The uppermost mantle shear wave velocity structure of eastern Africa from Rayleigh wave tomography: Constraints on rift evolution. Geophysical Journal International.

[49] Koptev A, Calais E, Burov E, Leroy S, Gerya T (2018). Along-axis Variations of Rift Width in a Coupled Lithosphere-Mantle System, Application to East Africa. Geophysical Research Letters.

[50] Dixon TH, Stern RJ, Hussein IM (1987). Control of Red Sea rift geometry by Precambrian structures. Tectonics.

[51] Ligi Marco, Bonatti Enrico, Bortoluzzi Giovanni, Cipriani Anna, Cocchi Luca, Caratori Tontini Fabio, Carminati Eugenio, Ottolini Luisa, Schettino Antonio (2012). Birth of an ocean in the Red Sea: Initial pangs. Geochemistry, Geophysics, Geosystems.

[52] Manighetti I, Tapponnier P, Courtillot V, Gruszow S, Gillot PY (1997). Propagation of rifting along the Arabia-Somalia plate boundary: The gulfs of Aden and Tadjoura. Journal of Geophysical Research: Solid Earth.

[53] Gerya, T. V. *Introduction to Numerical Geodynamic Modelling*. Cambridge University Press, p. 345 (2010).

[54] Duretz T, May DA, Gerya TV, Tackley PJ (2011). Discretization errors and free surface stabilization in the finite difference and marker-in-cell method for applied geodynamics: A numerical study. Geochemistry, Geophysics, Geosystems.

[55] Kohlstedt DL, Evans B, Mackwell SJ (1995). Strength of the lithosphere: constraints imposed by laboratory experiments. Journal of Geophysical Research: Solid Earth.

[56] Ranalli, G. Rheology of the Earth. Chapman and Hall, London, p. 413 (1995).

[57] Bürgmann R, Dresen G (2008). Rheology of the lower crust and upper mantle: evidence from rock mechanics, geodesy, and field observations. Annual Review of Earth and Planetary Sciences.

[58] Burov E, Cloetingh S (2010). Controls of mantle plumes and lithospheric folding on modes of intra-plate continental tectonics: differences and similarities. Geophysical Journal International.

[59] Burov E (2011). Rheology and strength of the lithosphere. Marine and Petroleum Geology.

