# Supplementary material for: Afar triple junction triggered by plume-assisted bi-directional continental break-up
Source: Sci Rep. 2018 Oct 3;8:14742. doi: 10.1038/s41598-018-33117-3 (PMC6170478; doi:10.1038/s41598-018-33117-3)
Supplement: Supplementary file 1 — Supplementary information [file 41598_2018_33117_MOESM1_ESM.docx]

**Afar triple junction triggered by plume-assisted bi-directional continental break-up**

Alexander Koptev^1,2^, Taras Gerya^3^, Eric Calais^4^, Sylvie Leroy^1^, and Evgueni Burov^1Ϯ^

^1^Sorbonne Université, CNRS, Institut des Sciences de la Terre de Paris (ISTeP), Paris, France ^2^Department of Geosciences, University of Tübingen, Tübingen, Germany

^3^ETH-Zurich, Institute of Geophysics, Sonnegstrasse 5, 8092 Zurich, Switzerland

^4^Ecole Normale Supérieure, Dept. of Geosciences, PSL Research University, CNRS UMR 8538, Paris, France

^Ϯ^ Deceased 9 October 2015

**SUPPLEMENTARY TABLES**

**Supplementary Table 1. Controlling parameters and resulting deformation style of 3D experiments.**

| *Experiment title* | *Controlling parameters* | | *Deformation mode* | *Supplementary Figure* |
| --- | --- | --- | --- | --- |
|  | *EW extension, V_ext_ (mm/yr)* | *Northward pull,*  *V_pull_ (mm/yr)* |  |  |
| Model **1** | 0 | 0 | axisymmetric deformation | Suppl. Fig. 2m |
| Model **2** | 0 | 4 | EW linear rift | Suppl. Fig. 2n |
| Model **3** | 0 | 0-6 | WNW-ESE linear rift | Suppl. Fig. 2o |
| Model **4** | 0 | 3-6 | WNW-ESE linear rift | Suppl. Fig. 2p |
| Model **5** | 0 | 6-12 | EW linear rift | Suppl. Fig. 2q |
| Model **6** | 0 | 6-18 | EW linear rift | Suppl. Fig. 2r |
| Model **7** | 3 | 0 | NS linear rift | Suppl. Fig. 2g |
| Model **8** | 3 | 4 | NS linear rift | Suppl. Fig. 2h |
| Model **9** | 3 | 0-6 | NS and NW-SE linear rifts | Suppl. Fig. 2i |
| Model **10** | 3 | 3-6 | triple junction | Suppl. Fig. 2j |
| Model **11** | 3 | 6-12 | WNW-ESE linear rift | Suppl. Fig. 2k |
| Model **12** | 3 | 6-18 | EW linear rift | Suppl. Fig. 2l |
| Model **13** | 6 | 0 | ridge-transform pattern | Suppl. Fig. 2a |
| Model **14** | 6 | 4 | NS linear rift | Suppl. Fig. 2b |
| Model **15** | 6 | 0-6 | NS linear rift | Suppl. Fig. 2c |
| Model **16** | 6 | 3-6 | ridge-transform pattern | Suppl. Fig. 2d |
| Model **17** | 6 | 6-12 | triple junction | Suppl. Fig. 2e |
| Model **18** | 6 | 6-18 | four interconnected triple junctions | Suppl. Fig. 2f |

**SUPPLEMENTARY FIGURES**


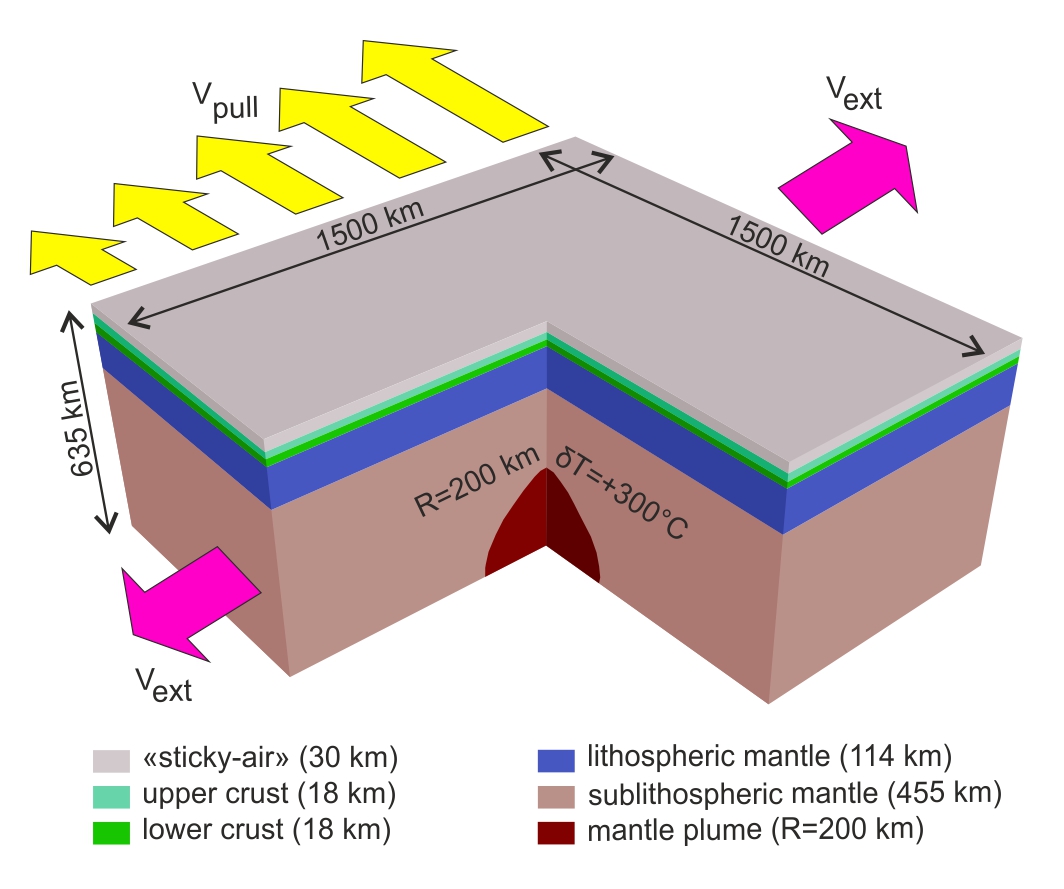


**Supplementary Figure 1.** Model setup. Symmetrical EW extension (V_ext_) and laterally varying northward pull (V_pull_) are shown by green and blue arrows, respectively.

**
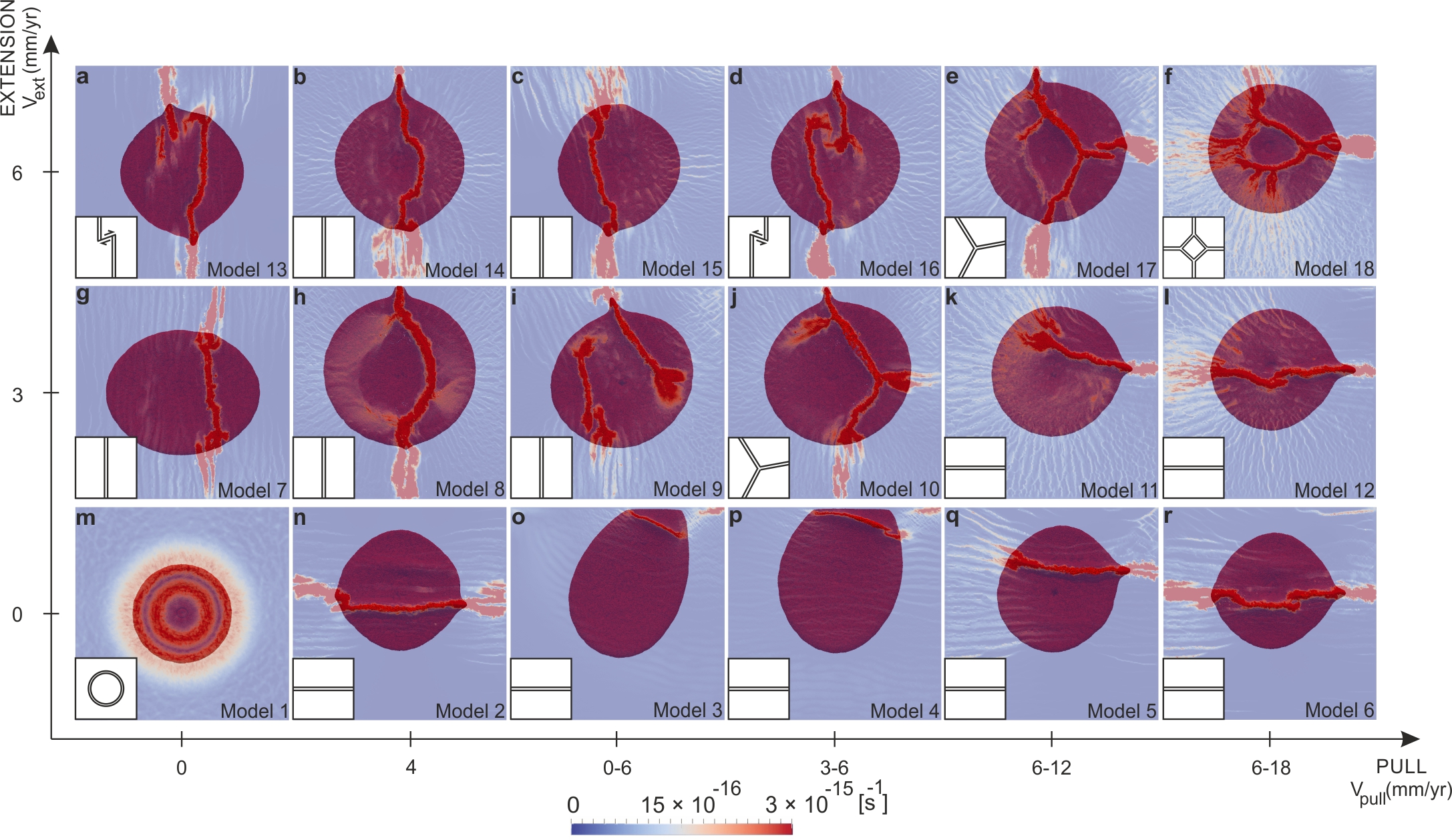
**

**Supplementary Figure 2.** Top view of all performed 3D experiments (see Supplementary Table 1). Blue to red colors indicate crustal strain rate at the level of 10 km (i.e. in the upper crust). The plume material is shown in dark red.


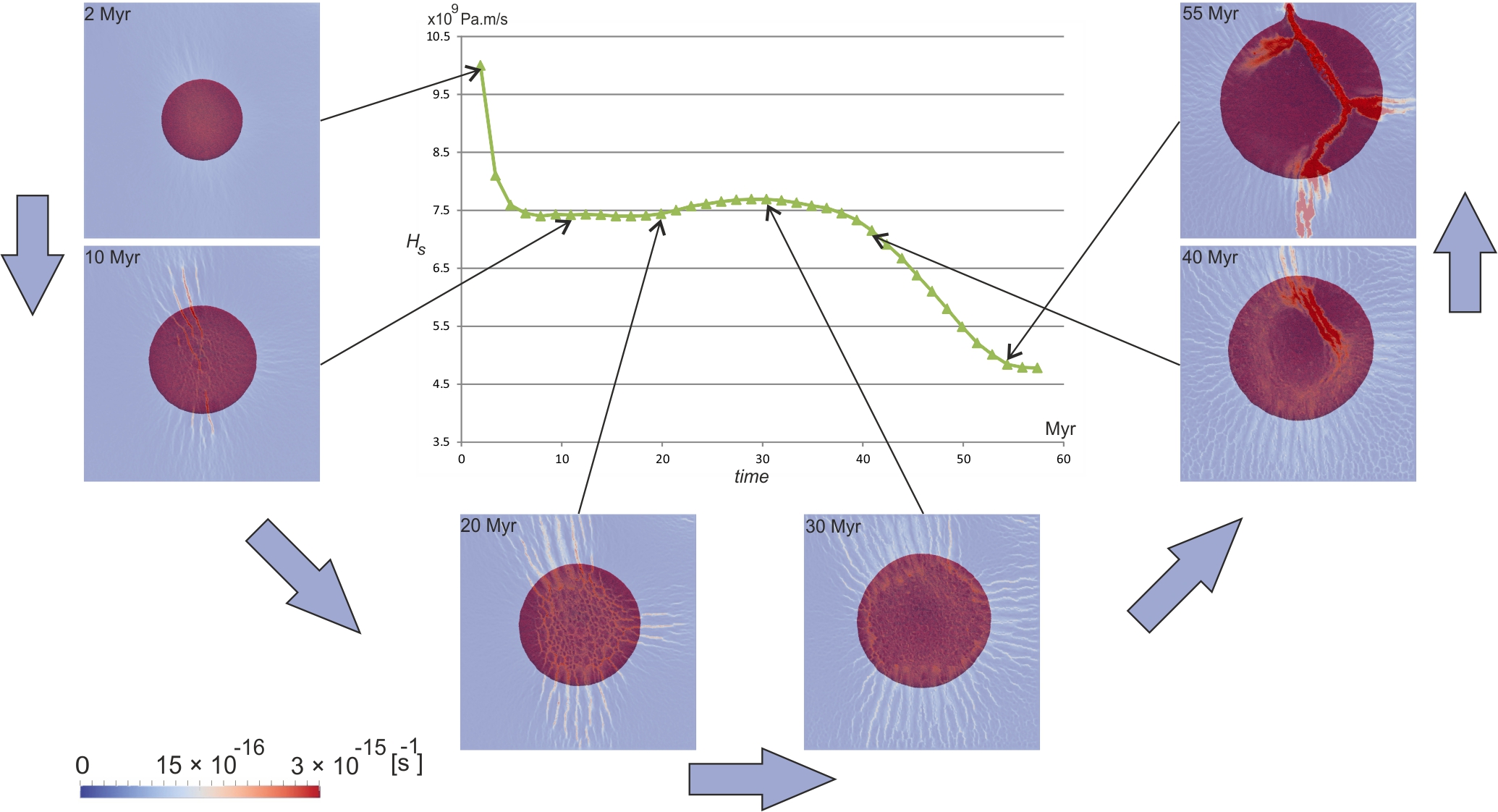


**Supplementary Figure 3.** Temporal evolution of triple junction model (see Fig. 2f; Fig. 3). The graph in center shows the shear heat production (*H_s_*) that is related to dissipation of the mechanical energy during irreversible non-elastic (e.g., viscous) deformation. Note that self-organized break-up geometry presumably maximizes the rate of decrease in the mechanical work needed for deformation. Total strain, localized on rift basins over the last 10-15 Myr of the model evolution, amounts to 40 km of total extension, in approximate accord with geological estimates from the Main Ethiopian rift (Corti, 2009) to Turkana (Hendrie et al., 1994). However, this agreement is not an accurate model validation given that the model parameters were not specifically tuned to match the details of tectonics observed in the studied area.

**References**

Corti, G. Continental rift evolution: from rift initiation to incipient break-up in the Main Ethiopian Rift, East Africa. Earth-Science Reviews **96**, 1–53 (2009).

Hendrie, D. B., Kusznir, N. J., Morley, C. K. & Ebinger, C. J. Cenozoic extension in northern Kenya: a quantitative model of rift basin development in the Turkana region. Tectonophysics **236**, 409-438 (1994).
